# Neuron devices: emerging prospects in neural interfaces and recognition

**DOI:** 10.1038/s41378-022-00453-4

**Published:** 2022-12-07

**Authors:** Yang Wang, Shuangjie Liu, Hao Wang, Yue Zhao, Xiao-Dong Zhang

**Affiliations:** 1 Tianjin Key Laboratory of Brain Science and Neural Engineering, Academy of Medical Engineering and Translational Medicine, Tianjin University, 300072 Tianjin, China; 2 Tianjin Key Laboratory of Low Dimensional Materials Physics and Preparing Technology, Institute of Advanced Materials Physics, School of Sciences, Tianjin University, 300350 Tianjin, China

**Keywords:** Neuron interface devices, Neural electrodes, Artificial sensory neuron devices, Brain-computer interfaces, Recognition, Electrical and electronic engineering, Electronic devices

## Abstract

Neuron interface devices can be used to explore the relationships between neuron firing and synaptic transmission, as well as to diagnose and treat neurological disorders, such as epilepsy and Alzheimer’s disease. It is crucial to exploit neuron devices with high sensitivity, high biocompatibility, multifunctional integration and high-speed data processing. During the past decades, researchers have made significant progress in neural electrodes, artificial sensory neuron devices, and neuromorphic optic neuron devices. The main part of the review is divided into two sections, providing an overview of recently developed neuron interface devices for recording electrophysiological signals, as well as applications in neuromodulation, simulating the human sensory system, and achieving memory and recognition. We mainly discussed the development, characteristics, functional mechanisms, and applications of neuron devices and elucidated several key points for clinical translation. The present review highlights the advances in neuron devices on brain-computer interfaces and neuroscience research.

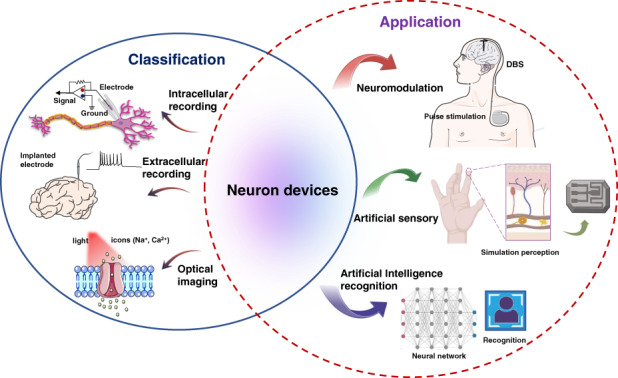

## Introduction

The nervous system has outstanding cognitive capabilities with 100 billion neurons, and the interrelationships among different types of neurons are extremely complex and precise^1^. A disorder of the nervous system poses a serious threat to human health; thus, developing high-level technologies and devices is very important for exploring brain function and understanding neuropathological progression; therefore, it has attracted great interest in the scientific community^2–8^. Correspondingly, neuron devices could improve the comprehension of neural networks and promote the diagnosis and treatment of nervous system diseases.

**Fig. 1:**
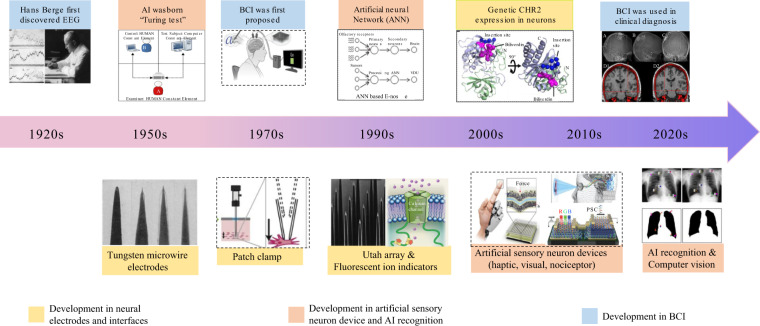
The development of neuron devices. The discovery of EEG signals in 1924. Reprinted from ref. ^12^. The design of the Turing test in 1950 to assess AI. Reprinted from ref. ^14^. The fabrication of tungsten microwire electrodes in 1957. Reprinted from ref. ^9^. Emergence of patch-camp technology in the 1970s. Reprinted from ref. ^10^. The proposal of BCI technology in 1977. Reprinted from ref. ^15^. ANN algorithm to realize classification and identification. Reprinted from ref. ^17^ . Genetically encoded CHR2 ion indicator expression in neurons. Reprinted from ref. ^11^. The development of artificial sensory neuron devices, including haptic, visual, and nociceptor devices, in the 2020s. Reprinted from refs. ^21,26^. The development of BCI clinical diagnosis and AI computer visual recognition in the 2020 s. Reprinted from refs. ^17,20^.

The development process of neuron devices is shown in Fig. 1. After the first discovery of electroencephalography (EEG) signals in the 1920s, neural electrodes and a variety of different signal detection technologies were gradually developed^9–13^. The Turing test sparked a wave of artificial intelligence in the 1950s, and researchers became increasingly interested in computer learning, gradually developing related technologies, such as deep learning and big data computing^14^. In the context of the continuous development of neuroscience and AI, brain-computer interface (BCI) technology was proposed in the 1970s^15^. To date, the cross-integration technology of artificial intelligence (AI) technology and brain science has boosted the development of neuron devices and neuroscience^16–18^. The BCI is currently in the stage of technological explosion, providing new neurorehabilitation methods and enabling disabled persons to control the external world by decoding the EEG signals obtained from neuron devices^15,19,20^. Furthermore, emerging neuron devices for simulating biological sensory neurons were invented to substitute disabled sense organs^21–26^. With the development of ‘big data’ technology, computer learning technologies based on neural networks have emerged and have shown great power to facilitate the development of neuron devices. Neuromorphic hardware and software systems simulating the plasticity of neurons and synapses can achieve memory and recognition^27,28^. However, some challenges, such as a low signal-to-noise ratio, immune response in neural tissue, unitary function and limited data processing capabilities^13,29–33^, have impeded the clinical translation of neuron devices. Therefore, it is of great significance to develop multifunctional neuron devices with high sensitivity, good biocompatibility and fast processing for the diagnosis and treatment of nervous system diseases.

In this review, we summarized representative neuron devices and their fabrication, properties, and biocompatibility; in addition, we highlighted their advances in biomedical applications. We analyzed the existing problems and challenges and elucidated future research directions, especially designing neuron devices combined with AI technology. First, we summarized the neuron interface devices that can sensitively monitor neuron firing signals in intracellular and extracellular space and in optical imaging. Second, we discussed the applications of neuron devices currently in development, including neuromodulation in neurological disorders, “sensory substitution” in motor prostheses, and memory and recognition in AI. We mainly discussed the development, characteristics, functional mechanisms, and application of neuron devices and elucidated several key points regarding clinical translation.

## Classification for neuron interface devices

High-quality neural signal recording requires sensitive signal acquisition devices, which can be combined with signal decoding to achieve self-feedback stimulation or control of the external world. The original intracellular neuron recording device was the patch-clamp technique, developed in the 1970s^34^, which detected synaptic transmission by manipulating high-temporal resolution electrical impulses on an individual neuron and was the gold standard for studying the properties of ion channels^35,36^. Afterward, integrated microelectrode arrays (MEAs) were developed to record large-scale neural activity to study communication between neuronal populations^30,37–40^.

However, foreign-body responses and inflammatory reactions can lead to the loss of neural signals in the electrode-nerve interface; thus, it is necessary to improve the biocompatibility of electrodes^13,30,41^. Other emerging neuron signal technologies, such as optogenetic modulation of neural activity, use optical stimulation, and imaging techniques with fluorescent indicators or genetically encoded molecular probes to enable large-scale recordings of neural activity^42,43^. In this section, we review the method of recording signals for neuron interface devices.

### Neuron interface devices for intracellular recordings

**Fig. 2:**
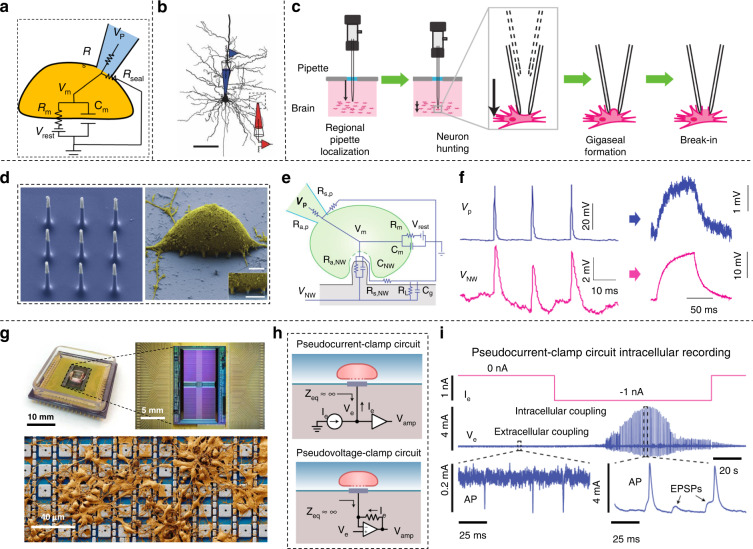
Neuron interface devices for intracellular recordings. **a** Schematic diagram of the working mechanism of the patch clamp. V_p_ represents the signal recorded by the pipette. Reprinted from ref. ^45^. **b** The patch clamp in the locations of the somatic (blue) and basal dendritic electrodes. Scale bar, 100 μm^47^. Copyright 2007 Springer Nature. **c** The algorithm of automatic patch-clamp in four stages: ‘localization’, ‘neuron hunting’, ‘gigaseal formation’, and ‘break­in'^50^. Copyright 2012 Springer Nature. **d** SEM image of VNEA and a rat cortical cell. Scale bar, 2.5 μm. **e** Equivalent-circuit model of the VNEA/cell interface. **f** The measurement of VNEA agreed with those obtained by patch pipette^51^. **g** A photograph of CNEI and false-color scanning electron microscope images of neurons cultured on CNEI. **h** Simplified signal model of the electrode and neuron interface for the pCC and pVC configurations. **i** Extracellular recordings transform into intracellular recordings by triggering an action potential in the pCC configuration^52^. Copyright 2020 Springer Nature.

Intracellular recording remains the best technique for capturing single-neuron electrical properties that contain crucial information regarding membrane ion-channel activities, receptor channel interactions, etc. The patch-clamp technique, which establishes direct contact with the intracellular environment through the penetration of a glass micropipette (Fig. 2a)^44–46^, is the most sensitive approach to investigating neural excitability. Thomas et al. investigated the propagation of axosomatic action potentials (APs) and postsynaptic potentials (EPSPs) transmitted to the basal tree with the patch-clamp technique, demonstrating that EPSPs are of prime importance to neuronal output (Fig. 2b)^47^. Kanako et al. used the patch-clamp technique to study firing patterns of dopamine neurons by recording the subthreshold potentials in vivo^48^. Nevertheless, this approach is restricted to single cells or channels and requires a high technical capability to perform. An automated patch clamp has the advantages of high throughput, ease of operation, and parallel detection of numerous cells^49^. Suhasa et al. developed a robot that automatically performed patch clamping and lowered its micropipettes until a cell was detected (Fig. 2c). Automated intracellular recording has the characteristics of good yield, throughput and quality^50^.

However, the patch-clamp technique is invasive and not suited for parallel execution of high-sensitivity intracellular recording for tens of minutes. Recently, emerging nanofabrication techniques have realized a large number of neuron intracellular recordings by developing nanoscale devices. A nanowire electrode array (VNEA) was developed, which can intracellularly record and stimulate cultured rat cortical neurons, demonstrating biocompatibility and biosafety (Fig. 2d–f)^51^. However, this kind of intracellular interface is significantly limited compared to that of the patch clamp and is inapplicable to neural networks. Electrical signal transmission between neural networks is precise and complex, and scalable and high-fidelity recording of intracellular signals from a large number of neurons is needed. A scalable recording composed of thousands of platinum-black electrodes, which can record intracellular electrical signals by stimulating a high-density neuron neuroelectronic interface (CNEI; Fig. 2g), was reported^52^. The CNEI simulated the working mechanism of the patch clamp setup and can operate in either pseudocurrent-clamp (pCC) mode or pseudovoltage-clamp (pVC) mode; this approach can be used to record membrane potential and ion channel currents by switching the two stimulation modes (Fig. 2h). Extracellular recordings of neurons were converted to intracellular measurements when given corresponding electrical stimulation (Fig. 2i). These results demonstrated that CNEI can be used to effectively perform the intracellular recording of single neuron firing and control neuronal spontaneous firing, which is important in neurological disorders caused by the high-frequency firing of spontaneous neurons^53,54^.

### Neuron interface devices for extracellular recordings

**Fig. 3:**
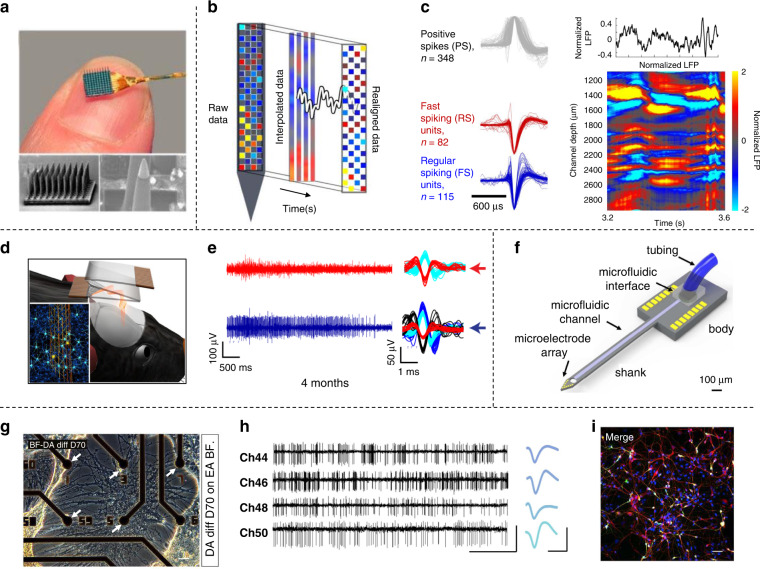
Neuron interface devices for extracellular recordings. **a** Prototype and SEM image of a Utah array. Reprinted from ref. ^56^. **b** Scheme of a Neuropixels probe. **c** The average waveforms of each unit neuron and average LFP along the depth of the Neuropixels probe^57^. Copyright 2022 Springer Nature. **d** Schematic of a mouse with stereotaxically injected mesh electronics (gold). **e** Single-unit recordings along with sorted spikes of one hemisphere (red traces) and the contralateral hemisphere (blue traces)^58^. Copyright 2016 Springer Nature. **f** Diagram of a multifunctional neural probe with drug delivery capability^59^. Copyright 2012 Royal Society of Chemistry. **g** Images of mature dopaminergic neurons cultured on MEA. **h** Four representative channels of spontaneous electrical activities. **i** Immunocytochemistry images of mature dopaminergic neurons cultured on the surface of MEA^60^. Copyright 2022 Elsevier.

Extracellular recording techniques are good for identifying high-frequency APs from single units and low-frequency local field potentials (LFPs) from groups of neurons^55^. The traditional Utah array can extend the sampling volume laterally (Fig. 3a), but such rigid probes can cause intrinsic tissue damage^56^. The electrode size was later reduced to be closer to that of the neuronal soma in the NeuroGrid array, which was an electrocorticography (ECoG) array. Recent advances in silicon Neuropixels probes have enabled large-scale neural recordings (Fig. 3b)^57^. The corresponding APs and average LFP were collected in the human cortex, suggesting that a Neuropixels probe could be adapted in acute recordings with high spatial sampling and high-quality spike sorting (Fig. 3c)^57^. To address the problem of the large difference between the bending stiffness of the neural probe and the Young’s modulus of neural tissue, mesh electronics, which achieved long-term recording of ~4 months (Fig. 3d, e)^58^, were designed. The biocompatibility of the electronic mesh was determined by exploring the number of astrocytes and microglia, which were naturally distributed around the electronic mesh, revealing less inflammation and steady periodic spike amplitudes.

Multifunctional neural probes integrated with diverse stimulation modalities, such as electrical, optical, and chemical stimulation, are becoming essential tools in neuroscience. Multifunctional probes with a microfluidic channel can deliver different neuroactive biochemicals and simultaneously monitor single-unit activities (Fig. 3f)^59^. In addition, the detection of electrophysiology combined with neurotransmitters, such as dopamine (DA), is expected to be a potential therapy for treating neurodegenerative diseases (Fig. 3g)^60^. Four representative channels of multifunctional MEA could be used to record electrical activities from mature dopaminergic neurons cultured on the surface of MEA (Fig. 3h). Fluorescence images showed that dopaminergic neurons cultured on MEA could highly express specific markers of mature dopaminergic neurons, demonstrating high biocompatibility and biosafety (Fig. 3i)^60^.

### Neuron interface devices for optical imaging recordings

**Fig. 4:**
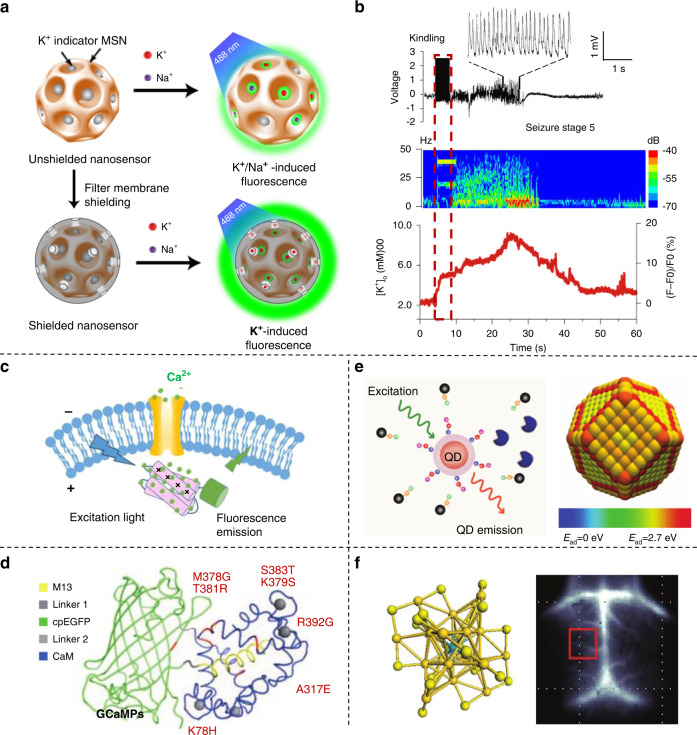
Neuron interface devices for optical imaging recordings. **a** Scheme of K^+^ nanosensors, which are especially sensitive to K^+^. **b** Electroneurographic signal and fluorescent imaging of a seizure stage mouse^69^. Copyright 2020 Springer Nature. **c** Schematic of genetically encoded calcium indicators^70^. Copyright 2013 Springer Nature. **d** The structure of the fluorophore sensor GCaMPs^71^. Copyright 2022 American Chemical Society. **e** Scheme of nanoparticle biosensors^77^. Copyright 2014 Science. **f** The structure of the Au cluster and brain imaging of a healthy mouse postinjection^75^. Copyright 2019 John Wiley & Sons.

Optical imaging and fluorescent probes provide powerful tools for the real-time detection of ion dynamics, which makes use of light as a sensor, providing high spatial resolution and avoiding electrical wire connections to tissue^61,62^. Changes in the extracellular potassium ion (K^+^) concentration affected the emission of nanosensors, which significantly regulated the potential, excitability, and spikes of cell membranes^63–65^. Extracellular K^+^ diminished the current driving power created by the activated K^+^ channel, resulting in a longer action potential duration and continuous excitation of neurons^66,67^. Some sensors based on biological components, such as vesicles, use membrane channel proteins to specifically transport K^+^ to indicators^68^. Some modified nanoparticle sensors, such as upconversion nanoparticle sensors, can convert near-infrared light to ultraviolet light, which triggers the detection of K^+^ fluctuations^61^. In addition, a highly sensitive and selective K^+^ sensor was designed by integrating commercially available K^+^ indicators into mesoporous silica nanoparticles (Fig. 4a)^69^. The recordings of epileptic mice correlated with the fluorescence signals during seizures showed that K^+^ nanosensors can noninvasively monitor electrical activity in freely moving mice (Fig. 4b)^69^.

Moreover, calcium (Ca^2+^) indicators have good compatibility with fluorescence microscopy, and genetically encoded indicators with fluorescent proteins have increased brightness and sensing accuracy (Fig. 4c)^70^. For example, GCaMPs have been frequently employed in in vivo research to discuss behavior-induced activities (Fig. 4d)^70,71^. The temporal response of calcium indicators is much slower than that of action potentials, so the indirect measurements of action potentials by calcium peaks sometimes do not provide a clear interpretation of the data^72^. Furthermore, nanomaterials, such as nanoparticles (NPs) and nanocluster-based sensors, enable efficient development of robust imaging probes for quantitative ion detection^73–76^. Colloidal nanoparticles with biocompatibility, strong fluorescence, long emissive lifetimes, and excellent photostability make them advanced biological sensors (Fig. 4e)^77^. Atomic-precision gold nanoclusters were designed to monitor high-resolution imaging under excitation of the long wavelength in near-infrared II (Fig. 4f)^32,75^. A clear blood vessel on brain images showed gold clusters with ultrasmall hydrodynamic sizes, exhibiting better resolution, which may be useful for future neuroscience applications.

## Applications of neuron devices

### Neuromodulation

**Fig. 5:**
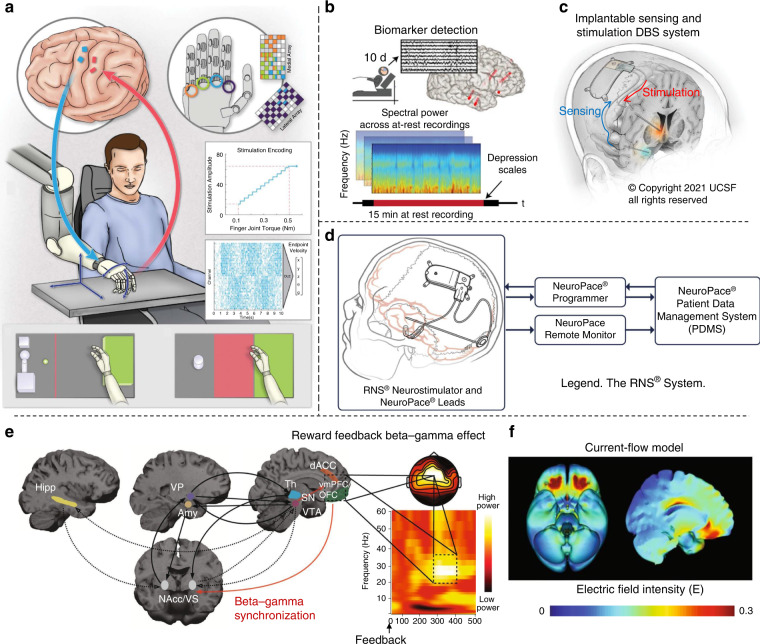
Neural electrical activity recording is applied to neuromodulation. **a** The overview of the bidirectional BCI system includes implanting microelectrodes, controlled robotic prosthesis, stimulation encoding, etc^78^. Copyright 2021 Science. **b** The recorded neural activity continuously for 10 days and the overall approach for biomarker detection. **c** Fully implantable DBS system^81^. Copyright 2021 Science. **d** The components of the RNS system include a neurostimulator and leads for recording and stimulation^82^. Copyright 2019 Elsevier. **e** Model integrating beta-gamma activity with reward learning circuitry. **f** Current-flow models on three-dimensional reconstructions of the cortical surface^84^. Copyright 2021 Springer Nature.

The modern era of neuromodulation began in the early 1960s and refers to electrical stimulation or chemical substances applied directly to the nervous system to modify nerve cell activity. The applications of therapeutic electrical stimulation are very diverse, and new applications are being developed. In recent years, with the development of artificial intelligence technology, brain-computer interface (BCI) technology holds great potential as a neuromodulation tool for helping patients with neuromotor dysfunction. Recently, a developed bidirectional BCI system was shown to control robotic prostheses in real time through signal monitoring of the implanted microelectrode array (Fig. 5a)^78^, which can evoke tactile sensations by stimulating the motor cortex and decoding neural recordings to control the prosthesis. In the future, systematic BCI technology also needs to be designed with a more complete stimulus encoding and decoding system, which will promote somatosensory recovery in patients with motor dysfunction.

Sensing and feedback are two functions of closed-loop regulation that can rapidly and consistently improve the treatment of neurological conditions^79^. Recent studies have shown that deep brain stimulation (DBS) combined with closed-loop strategies can treat Parkinson’s disease and other motor disorders^80^. Scangos et al. developed an approach to implement depression-specific biomarker-driven closed-loop therapy by implanting a chronic deep brain sensing and stimulation device (Fig. 5b)^81^. Such a device designed with multichannel recording, biomarker detection and microstimulation technologies was able to reduce the frequency of seizures with safety and good tolerance (Fig. 5c, d)^81,82^. In the future, there is a need for further development of the loop for DBS, which is wireless, compact, robust, and biocompatible. In addition, noninvasive neuromodulation, such as transcranial alternating current stimulation (tACS), can intervene with neurophysiological dynamics^83^. Grover et al.^84^ used high-frequency tACS to establish beta-gamma rhythms in reward learning for obsessive-compulsive disorder (OCD) (Fig. 5e). The underlying mechanism of OCD was verified, and the corresponding current-flow model of the cortical surface was reconstructed in three dimensions (Fig. 5f). These noninvasive techniques also include transcranial magnetic stimulation and focused ultrasound, which provides insight into brain physiology and is used to modify brain circuits for various therapeutic and neural enhancements.

### Artificial sensory neuron devices

**Fig. 6:**
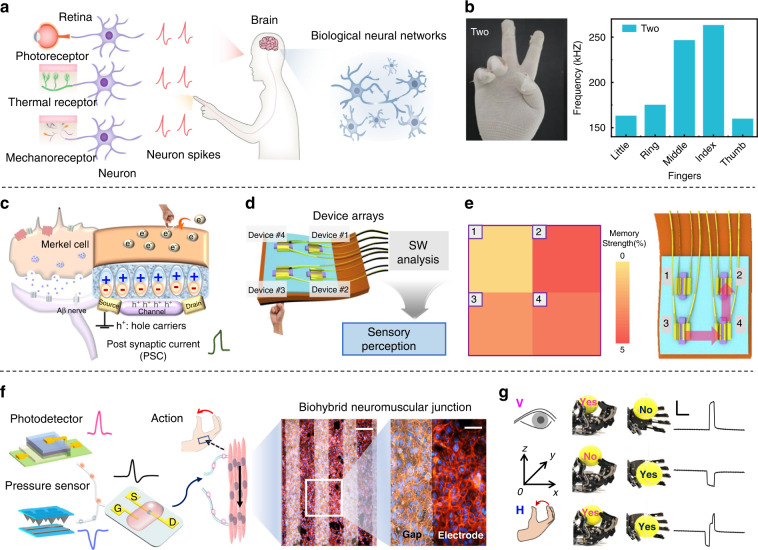
Artificial neuron sensory devices for mimicking the human sensory system. **a** Scheme of the biological perception system. **b** Response of artificial spiking curvature sensory neurons to the gesture ‘Two’ and corresponding statistics of spiking frequency^91^. Copyright 2022 Springer Nature. **c** Schematic diagram of the electronic structure corresponding to a Merkel cell. **d** Schematic of the AiS-TSO reception and processing simultaneously and intelligent memory. **e** Diagram of the corresponding relationship between the pixel values and the expected order according to SW analysis. Reproduced with permission^93^. Copyright 2020 Springer Nature. **f** Schematic of visual-haptic fusion controlling muscle action and imaging of neuromuscular junction. **g** Visual-haptic fusion based on BASE for manipulator control robot arms^97^. Copyright 2020 Springer Nature.

In biological perception systems, certain types of neurons and receptors, such as photoreceptors and mechanoreceptors, transform external environmental signals into electrical spikes (Fig. 6a)^85–91^. Artificial sensory neuron devices can mimic complicated sensing and processing functions in biological systems, which can convert external stimuli into electrical signals. Recently, emerging devices, such as memristors, have been used to emulate the functionalities of synapses and neurons. Yuan et al.^91^ reported a neuromorphic perception system that can monitor the curvatures of fingers by using the perception component VO_2_ (Fig. 6b). In addition, Bao et al. created an electronic pressure sensor with neuron-like devices using flexible degradable materials, which can move to monitor electrocardiogram and electromyogram signals^92^. Shun et al. reported an artificial haptic sensory system that can simulate fast adaptation and slow adaptation by stress and vibration and that can then output physiological signals^21^. In addition, an artificial intrinsic-synaptic tactile sensory organ (AiS-TSO) was developed, which mimicked synaptic connections and had sensory and memory functions^93^. The sensing mechanism was the influx of Ca^2**+**^ induced by Merkel cells under pressure (Fig. 6c)^94^, which realized the memory function of the synapse, and the order of touches can be deduced by the size of the pixel values of the device array (Fig. 6d, e). Therefore, the simulated tactile receptor with simple memory and recognition functions can flexibly control the reception and processing of tactile information.

Human perception of the external environment is extremely complex and depends on the fusion of multiple senses. The fusion and connection of multiple sensors enables the brain to perform cognition correctly^95^. For example, using innovative materials and technologies resulted in efficient and sensitive monitoring of sensory information, reaching the level of human receptors^96^. A bimodal artificial sensory neuron (BASE) realized the fusion of visual and haptic modalities^97^. Pressure sensors and photodetectors were the major components of the BASE patch, which operated as receptors in the retina and skin, respectively, transforming tactile and visual stimuli into electrical impulses. Signals transmitted from the BASE patch acted on skeletal myotubes through constructed neuromuscular junctions to simulate muscle motor control (Fig. 6f). Both visual feedback and tactile feedback were used to create the movement of a robot’s hands. However, merely supplying one-dimensional information led to placement problems, and the two modes cooperated to enable the robot arm to grip the target more accurately (Fig. 6f, g). Artificial sensory neurons/synapses with a fusion of touch and vision have been used in applications such as pattern recognition and postural control, but the perception of reliability, sensitivity, and accuracy of these mechanisms need to be improved. The artificial sensory system provides significant technical support and a driving force for biomedical and engineering application research and provides a bright future for the creation of intelligent prosthetics, intelligent organs, and humanoid robots.

### Artificial intelligence memory and recognition

**Fig. 7:**
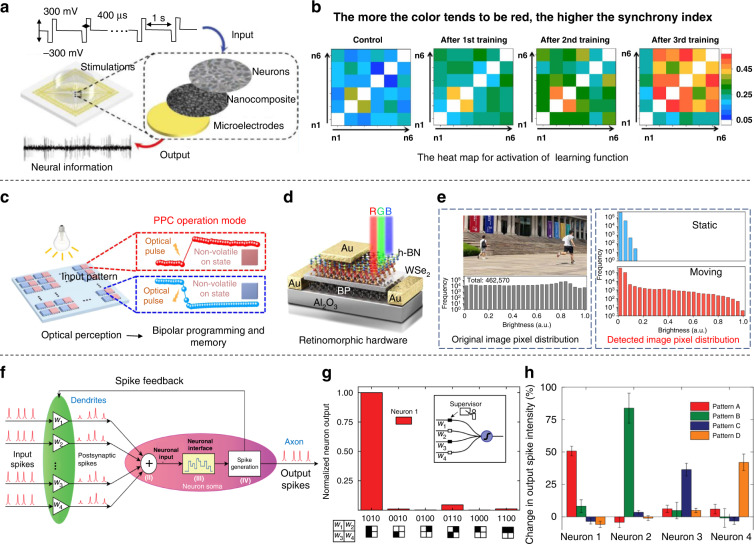
Artificial intelligence memory and recognition. **a** Scheme of high-throughput MEA for recording and stimulation. **b** The process of dynamic changes in the synchrony index of the network^103^. Copyright 2022 American Chemical Society. **c** Scheme of retina-inspired 2D hardware for simulating photoreceptors and memory functions. **d** Schematic diagram of the device structure and material composition. **e** Original image and normalized pixel distribution image for motion recognition of the hardware^105^. Copyright 2021 Springer Nature. **f** Schematic diagram of neurosynaptic network hardware simulating neurons and synaptic connections. **g** An individual neuron on the neurosynaptic chip achieved supervised learning. **h** A device consisting of four photonic neurons performing the recognition function of a neural network. Reproduced with permission^107^. Copyright 2019 Springer Nature.

Neuroscientists are now paying more attention to the brain’s learning and memory functions^98,99^. Regarding the mechanisms of learning and memory mediated by neural networks, the current electrophysiological detection and regulation technology at the cellular level is not enough^22,100–102^. A high-performance electronic device was designed to train hippocampal neurons to learn by activating their memory function through electrical stimulation (Fig. 7a, b)^103^. Correlation and synchrony of the hippocampal neuronal networks with training were examined by a heatmap, which showed that the synchrony index increased with increasing training time (Fig. 7b). Furthermore, based on retinal photoreceptors and bipolar cells for motion detection and recognition (MDR), the two-dimensional retinal neuron hardware integrated three modules of optical perception, memory, and recognition^104,105^. The nonvolatile positive photocurrent (PPC) and negative photocurrent (NPC) matched the photoconductive switching states of the simulated retina photoreceptor and bipolar cell processing memory, realizing the detection and memory of moving objects (Fig. 7c, d)^106^. The motion detection function showed that the normalized pixel brightness of the static object was approximately zero, while the pixel brightness of a moving object was distributed in the whole region (Fig. 7e). This MDR hardware that was developed to simulate the function of the human retina can achieve efficient recognition and memory functions, which greatly promoted retinal simulation technology.

In addition, an all-optical pulse neuron device was designed to accomplish the AI task of pattern recognition^107^. A light pulse entered from the presynaptic neuron, forming postsynaptic spikes after certain weight processing, which was transmitted to a postsynaptic neuron (Fig. 7f). Neuron circuit components were applied to realize the function of AI^108^. In supervised mode, the device correctly learned and recognized “1010” and trained successfully when the neuron was subjected to input mode (Fig. 7g). The results showed that the neurosynaptic network circuit simulated synapses and pulse transmission between pairs of neurons using phase transitions of light-triggered material with the correct learning function. In addition, the neural network composed of four neurons showed different spike intensity changes after the four training modes, indicating that the designed neural network successfully recognized the four modes (Fig. 7h). The neural network simulated by this integrated design can self-learn to complete simple recognition tasks, and it runs several orders of magnitude faster than biological neural networks; thus, it can process large amounts of data in a short time. Deciphering the mechanisms of human memory is a major goal of neuroscience, and artificial intelligence memory and recognition could advance the treatment of memory disorders in humans.

## Conclusions

By imitating the intricate design and function of the brain, neuron devices were developed to probe neural networks. To better understand and utilize the functional mechanism of the nervous system, neural signals were combined with various applied devices to advance science and society. The fusion of AI technology and neuroscience will facilitate the development of neuron devices, which is a common concern for researchers and patients. Of note, the integration of the efficiency and biosafety of materials will become design criteria for neuron devices, and several challenges should be addressed before clinical use^109–111^.

**Fig. 8:**
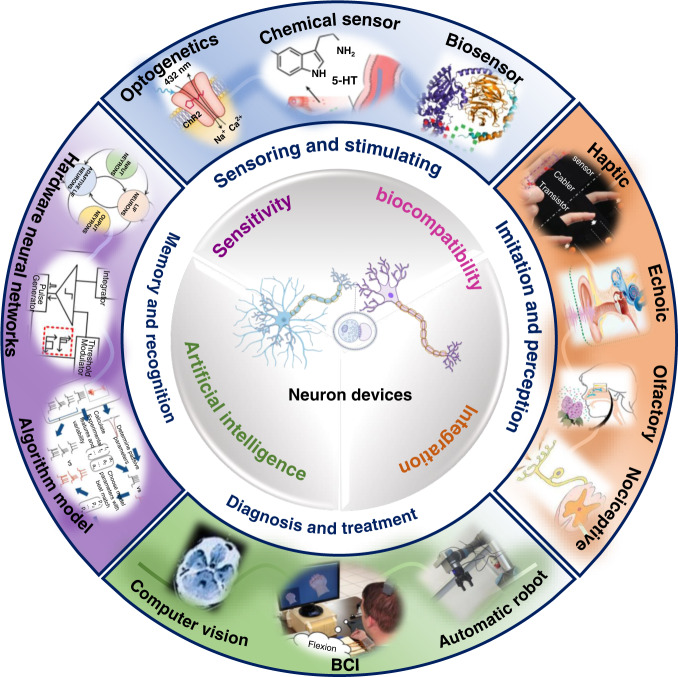
Future ptospects of neuron devices.

The design of neuron devices may focus on exploring a stable neural-electrode interface, an exquisite design process and efficient data processing. To develop ideal neuron devices, several aspects should be taken into account, as shown in Fig. 8. First, more sensitive neuron devices need to be developed to overcome the limitations of low sensitivity for neural signal recording^13,31,109–113^. The design of implant electrodes with smaller size and higher spatiotemporal resolution of sensors, such as optogenetic nanomaterials, biosensors and chemical sensors, may enable long-term sensitive signal recording^6,32,33,114–118^. Second, biocompatibility is important for the development of neuron devices. An electrode can be modified with nanomaterials possessing high catalytic activity, such as nanoclusters^114,119–123^, atomic level nanozymes^122,124–129^ and two-dimensional materials^130–133^, to reduce the inflammatory response of neural tissue. Third, integrated neuron devices can be developed to achieve multiple functions simultaneously^134–136^. For example, closed-loop monitoring-stimulation systems can be implanted into abnormal brain regions for long-term monitoring and treatment of neurological disorders, such as epilepsy and Alzheimer’s disease. Finally, AI technology can be used to achieve fast and efficient data processing. Combining AI with BCI and exploiting neural network algorithms will propel the development of neuron devices and improve neuroscience research.
